# Transcranial Direct Current Stimulation Does Not Affect Lower Extremity Muscle Strength Training in Healthy Individuals: A Triple-Blind, Sham-Controlled Study

**DOI:** 10.3389/fnins.2017.00179

**Published:** 2017-04-04

**Authors:** Kazuhei Maeda, Tomofumi Yamaguchi, Tsuyoshi Tatemoto, Kunitsugu Kondo, Yohei Otaka, Satoshi Tanaka

**Affiliations:** ^1^Tokyo Bay Rehabilitation HospitalChiba, Japan; ^2^Department of Rehabilitation Medicine, Keio University School of MedicineTokyo, Japan; ^3^Department of Neuroscience and Pharmacology, University of CopenhagenCopenhagen, Denmark; ^4^Department of Physical Therapy, Yamagata Prefectural University of Health SciencesYamagata, Japan; ^5^Laboratory of Psychology, Hamamatsu University School of MedicineShizuoka, Japan

**Keywords:** transcranial direct current stimulation, strength training, lower limb, primary motor cortex, rehabilitation

## Abstract

The present study investigated the effects of anodal transcranial direct current stimulation (tDCS) on lower extremity muscle strength training in 24 healthy participants. In this triple-blind, sham-controlled study, participants were randomly allocated to the anodal tDCS plus muscle strength training (anodal tDCS) group or sham tDCS plus muscle strength training (sham tDCS) group. Anodal tDCS (2 mA) was applied to the primary motor cortex of the lower extremity during muscle strength training of the knee extensors and flexors. Training was conducted once every 3 days for 3 weeks (7 sessions). Knee extensor and flexor peak torques were evaluated before and after the 3 weeks of training. After the 3-week intervention, peak torques of knee extension and flexion changed from 155.9 to 191.1 Nm and from 81.5 to 93.1 Nm in the anodal tDCS group. Peak torques changed from 164.1 to 194.8 Nm on extension and from 78.0 to 85.6 Nm on flexion in the sham tDCS group. In both groups, peak torques of knee extension and flexion significantly increased after the intervention, with no significant difference between the anodal tDCS and sham tDCS groups. In conclusion, although the administration of eccentric training increased knee extensor and flexor peak torques, anodal tDCS did not enhance the effects of lower extremity muscle strength training in healthy individuals. The present null results have crucial implications for selecting optimal stimulation parameters for clinical trials.

## Introduction

Transcranial direct current stimulation (tDCS) is a non-invasive cortical stimulation procedure in which weak direct currents polarize target brain regions (Nitsche and Paulus, 2000). The application of anodal tDCS to the primary motor cortex of the lower extremity transiently increases corticospinal excitability in healthy individuals (Jeffery et al., 2007; Tatemoto et al., 2013) and improves motor function in healthy individuals and patients with stroke (Tanaka et al., 2009, 2011; Madhavan et al., 2011; Sriraman et al., 2014; Chang et al., 2015; Montenegro et al., 2015, 2016; Angius et al., 2016; Washabaugh et al., 2016). Thus, anodal tDCS has a potential to become a new adjunct therapeutic strategy for the rehabilitation of leg motor function and locomotion following a stroke.

Lower leg muscle strength is an important motor function required for patients who have had a stroke to regain activities of daily living (ADL). Lower leg muscle strength correlates with performance in activities, including sit-to-stand, gait, and stair ascent (Bohannon, 2007). Furthermore, lower leg muscle strength training increases muscle strength and improves ADL in patients with stroke (Ada et al., 2006). Therefore, lower leg muscle strength training is one of the important activities rehabilitating patients with stroke to regain their independence in ADL.

Several studies have examined the effect of a single session of tDCS on lower leg muscle strength, although the evidence is inconsistent (Tanaka et al., 2009, 2011; Montenegro et al., 2015, 2016; Angius et al., 2016; Washabaugh et al., 2016). Its effects seem dependent on tDCS protocols, training tasks, muscle groups, and subject populations. Although, most tDCS studies on lower leg muscle strength have focused on the acute effects of a single tDCS application, to the best of our knowledge, no study has examined how lower extremity strength training combined with repeated sessions of tDCS affects lower leg muscle strength. This type of investigation has strong clinical implications for the application of tDCS in rehabilitation for patients with lower leg muscle weakness.

Thus, to examine whether anodal tDCS can enhance the effects of lower extremity muscle strength training, the present study simultaneously applied anodal tDCS and lower extremity muscle strength training to healthy individuals and evaluated their effects on lower extremity muscle strength.

## Methods

### Participants

Twenty-four healthy adult volunteers (12 men and 12 women; age [mean ± standard deviation], 23.7 ± 1.3 years) participated in the study (Table 1). The sample size was calculated using a power analysis with a test family = *F*-tests, statistical test = analysis of variance (ANOVA): repeated measures between factors, effect size of *d* = 1.02 (Tanaka et al., 2009), α error probability = 0.05, and β error probability = 0.10. The analysis gave a sample of 10 participants per group. Considering a 20% drop-out rate, 12 participants in each group (a total of 24 participants) were recruited in this study. From a statistical viewpoint, a minimum sample size of 12 participants per group is recommended for a pilot study (Julious, 2005). None of the participants had a history of neurological and/or orthopedic diseases or was being treated with any medication that affected the central nervous system. All participants gave their written informed consent before participating in the study. This study was approved by the Tokyo Bay Rehabilitation Hospital ethics committee (approval number: 37-3), and it was performed in accordance with the ethical standards laid down in the Declaration of Helsinki.

**Table 1:** **Participants' characteristics**.

**Variable**	**Anodal tDCS group (*n* = 12)**	**Sham tDCS group (*n* = 12)**	**95% CI**	***P*-value**
Age (years)	23.9 (1.3)	23.5 (1.4)	−0.7, 1.5	0.799
Sex, male/female (number)	6/6	6/6	NA	0.658
Height (cm)	165.6 (9.0)	165.3 (8.6)	−7.2, 7.7	0.916
Weight (kg)	57.8 (10.3)	56.6 (10.4)	−7.6, 9.8	0.870
Body mass index (kg/m^2^)	20.9 (2.1)	20.5 (1.9)	−1.3, 2.1	0.255
**KNEE EXTENSOR TORQUE (Nm) AT BASELINE**				
Intervention side	155.9 (45.3)	164.1 (54.7)	−50.8, 34.3	0.700
Non-intervention side	162.9 (56.0)	159.6 (53.0)	−42.9, 49.4	0.331
**KNEE FLEXOR TORQUE (Nm) AT BASELINE**				
Intervention side	81.5 (30.3)	78.0 (29.8)	−22.0, 28.9	0.634
Non-intervention side	81.8 (27.9)	86.3 (25.8)	−27.2, 18.3	0.676

*Data are presented as the mean ± standard deviation. 95% CI = 95% confidence interval of the difference between the means (anodal group—sham group), tDCS, transcranial direct current stimulation; NA, not applicable*.

### Experimental procedures

The study used a triple-blind (participants, outcome assessor, and data analyst), sham-controlled experimental design to minimize biased assessment of tDCS intervention effects. Participants were stratified by sex and were randomly allocated to receive anodal tDCS combined with lower extremity muscle strength training (anodal tDCS group) or sham tDCS combined with lower extremity muscle strength training (sham tDCS group). Muscle strength training was conducted once every 3 days for 3 weeks (7 sessions). Peak torques of knee extension and flexion were measured before and after the 3 weeks of training, and throughout the training sessions. Pre-training assessments were conducted between 72 and 48 h before the first training session. Post-test assessments were conducted between 48 and 72 h after the final training session.

### Muscle strength training

**Figure 1:**
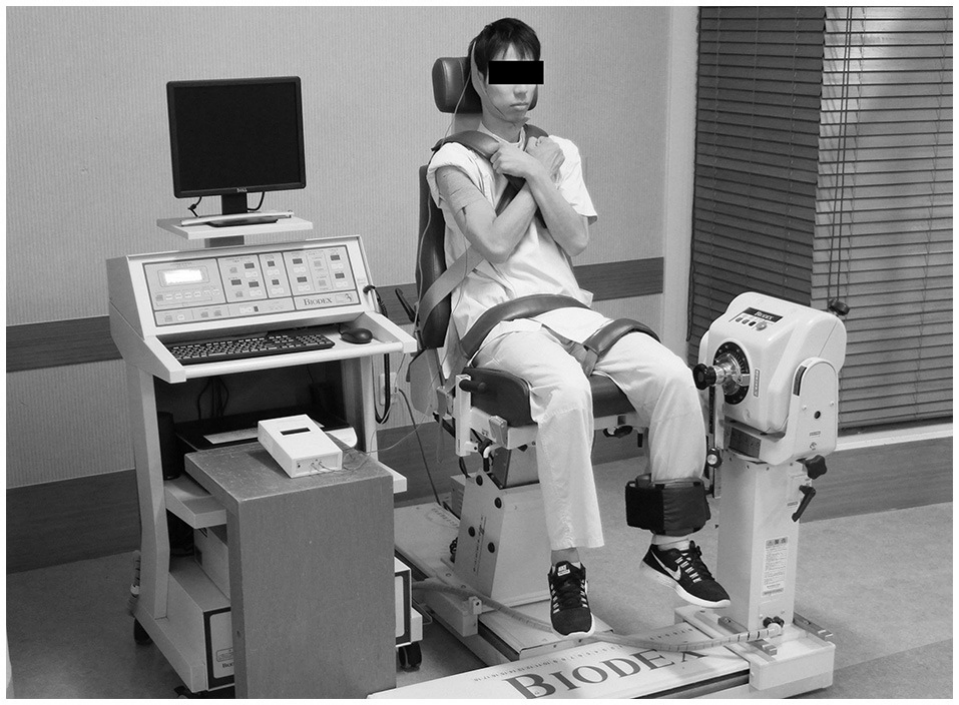
**Experimental setup of the muscle strength training and torque assessment**.

**Figure 2:**
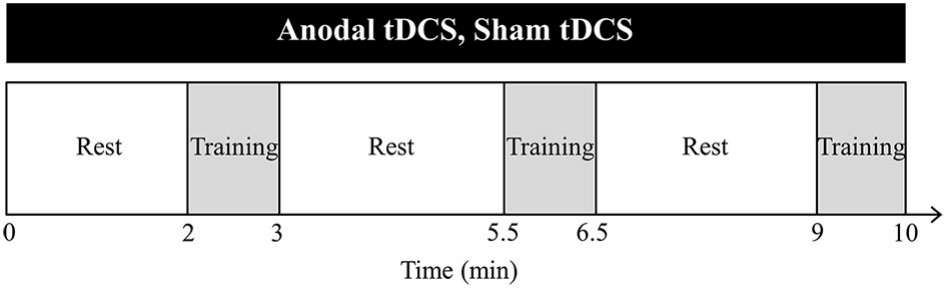
**Training protocol**. Anodal transcranial direct current stimulation (tDCS) (2 mA, 10 min) is applied to the primary motor cortex for the lower extremity during muscle strength training. In the sham tDCS experiment, the same procedure is used, but it is only delivered for ~15 s to mimic the transient skin sensation. Muscle strength training consists of 3 sets of 10 maximum isokinetic eccentric contractions on the knee extensors and flexors, with a 150-s rest period between sets. Training is performed once every 3 days for 3 weeks (7 sessions).

The setup for muscle strength training and torque measurement was shown in Figure 1. Participants underwent an eccentric training protocol focused on the knee extensors and flexors of their non-dominant side. Each participant's dominant leg was established using the Footedness Questionnaire (Chapman et al., 1987). An isokinetic dynamometer (Multi-Joint 3, Biodex Medical Systems, Inc., Shirley, NY, USA) was used as a training device. Participants were positioned with the backrest reclined 5° from vertical and the knee flexed at 90° (Figure 1). To avoid compensatory movements, straps were positioned across the participant's trunk, pelvis, and thigh. The dynamometer axis was aligned with the axis of rotation of the knee joint (lateral femoral epicondyle), and the dynamometer's lever arm was attached to the distal leg (above the medial malleolus). The knee was moved by the dynamometer through the range of motion from 20° to 90° of knee flexion (Ahmed et al., 2011). The training protocol consisted of 3 sets of 10 maximum isokinetic eccentric contractions at 30°/s (Poletto et al., 2008), with a 150-s rest period between sets (Figure 2). Participants were instructed to extend the knee with maximal effort while the dynamometer flexed the knee at 30°/s from 20° to 90° as eccentric training of the knee extensors. For the training of the knee flexors, participants flexed the knee while the dynamometer extended the knee at 30°/s from 90° to 20°. The training commenced 2 min after tDCS onset and ceased at the same time as tDCS offset. Participants performed this training once every 3 days for 3 weeks (7 sessions), with an interval of at least 48 h between sessions. Before and after each training session, participants warmed up on a stationary bicycle for 5 min, followed by performing a set of stretches focused on the knee extensor and flexor muscles.

### tDCS

Anodal tDCS (2 mA, 10 min) was delivered by a DC-Stimulator-Plus (NeuroConn, Ilmenau, Germany) connected to a pair of sponge-surface electrodes, each with a surface area of 25 cm^2^, soaked in a 0.9% NaCl saline solution. The anodal electrode was positioned over the non-dominant leg representation in the primary motor cortex, and the reference electrode was placed over the ipsilateral upper arm (Tatemoto et al., 2013; Angius et al., 2016). Although, tDCS studies using a cephalic reference electrode have reported significant effects on cortical excitability and performance (Jeffery et al., 2007; Tanaka et al., 2009, 2011; Madhavan et al., 2011; Sriraman et al., 2014; Chang et al., 2015; Washabaugh et al., 2016), the arrangement potentially created unwanted changes in frontal cortex excitability under the reference electrode (Moliadze et al., 2010; Vandermeeren et al., 2010). For this reason, we decided to use an extracephalic reference electrode in the present study. Previous tDCS studies with an extracephalic reference electrode have reported significant effects on lower limb cortical excitability and performance (Tatemoto et al., 2013; Angius et al., 2016). The position of the primary motor cortex was confirmed based on the induction of the largest motor evoked potentials in the rectus femoris muscle with a constant stimulus intensity using transcranial magnetic stimulation (TMS) with a double-cone stimulation coil connected to a Magstim 200 magnetic stimulator (Magstim, Whitland, UK). For the sham stimulation, the same procedure was performed, but the current was applied for only 15 s (Gandiga et al., 2006).

### Torque evaluation

Participants were positioned in an isokinetic dynamometer with the same settings that were used during training. After a familiarization period, consisting of five submaximal eccentric contractions of the knee extensors and flexors, maximal knee extensor and flexor torques were evaluated under eccentric (30°/s) conditions. The evaluation task comprised five consecutive maximal isokinetic eccentric contractions of knee extension and flexion. The peak torque was defined as the maximum value achieved during the five contractions. During the evaluations, examiners provided participants with verbal encouragement to apply their maximal effort.

### Data analysis

Data for each of the four muscle groups (non-dominant knee extensors, dominant knee extensors, non-dominant knee flexors, and dominant knee flexors) were separately analyzed. The primary outcome measure was the peak torque data before and after 3 weeks of training. For the peak torque of each muscle group, we applied a 2-factor ANOVA to evaluate the intervention (anodal and sham) and time (pre-training and post-training) as within-subject factors. The secondary outcome measure was the peak torque data on the intervention side during the training sessions. To evaluate the acute effects of tDCS on the peak torque, we used a 2-factor repeated-measures ANOVA with the intervention (anodal and sham) and time (7 sessions) as factors. Partial eta squared ($\eta_{p}^{2}$) was calculated as a measure of the effect size. Mean square error (MSe) was also presented. *Post-hoc* tests were performed using independent *t*-tests with Bonferroni correction for multiple comparisons. Statistical analyses were performed using IBM SPSS 22.0 (IBM Corp., Armonk, NY, USA). Statistical significance was defined as *p* < 0.05 for all comparisons.

## Results

All participants successfully completed training for 3 weeks. There were no reports of adverse events due to the training or tDCS.

**Figure 3:**
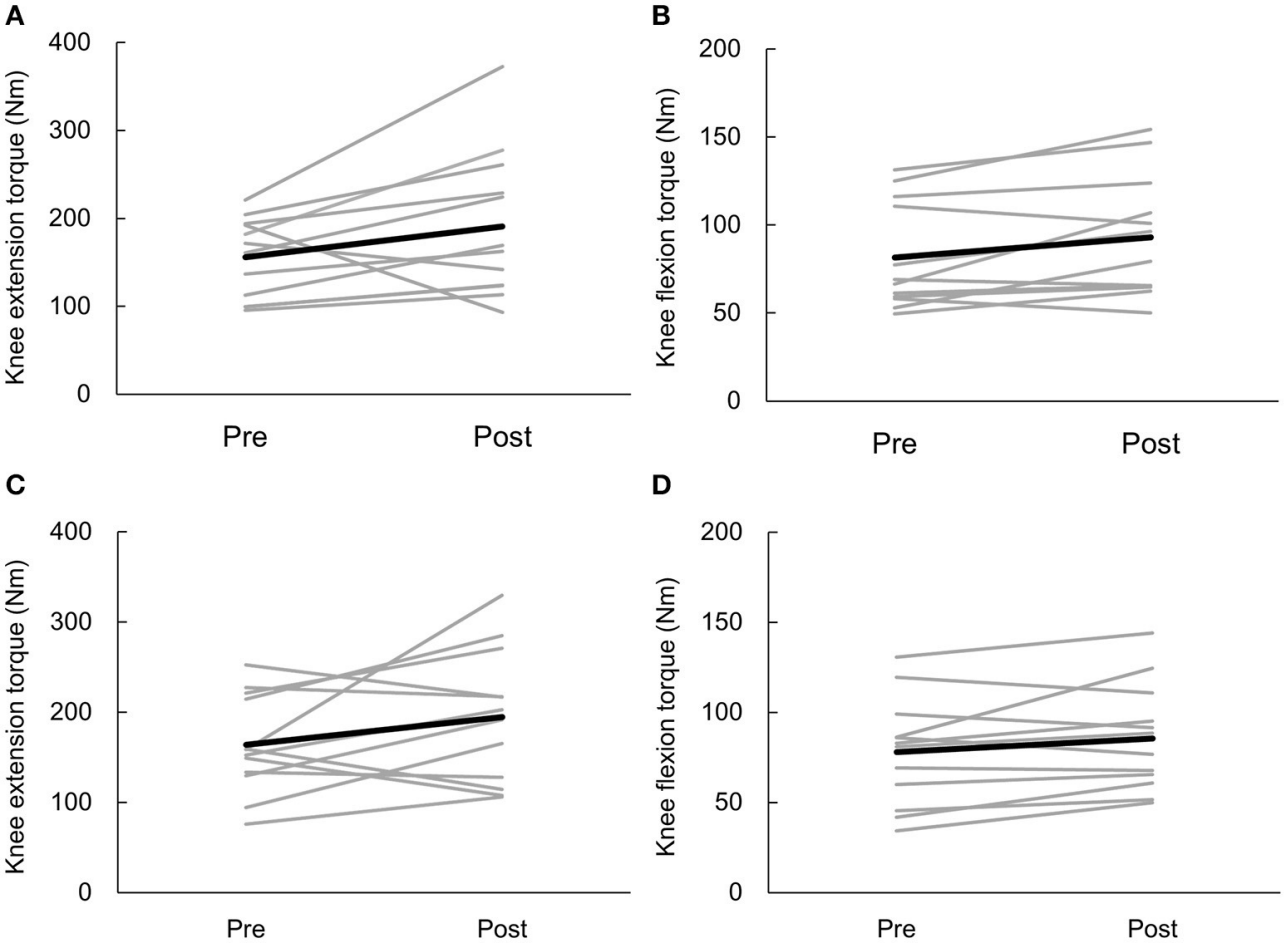
**Individual participant data of knee torque on the intervention side before (Pre) and after (Post) anodal transcranial direct current stimulation (tDCS) (real and sham)**. Black lines represent the mean data. Gray lines represent the individual participant data. Effects of anodal tDCS combined with muscle strength training on knee extensor torque **(A)** and knee flexor torque **(B)**. Effects of sham tDCS combined with muscle strength training on knee extensor torque **(C)** and knee flexor torque **(D)**.

**Figure 4:**
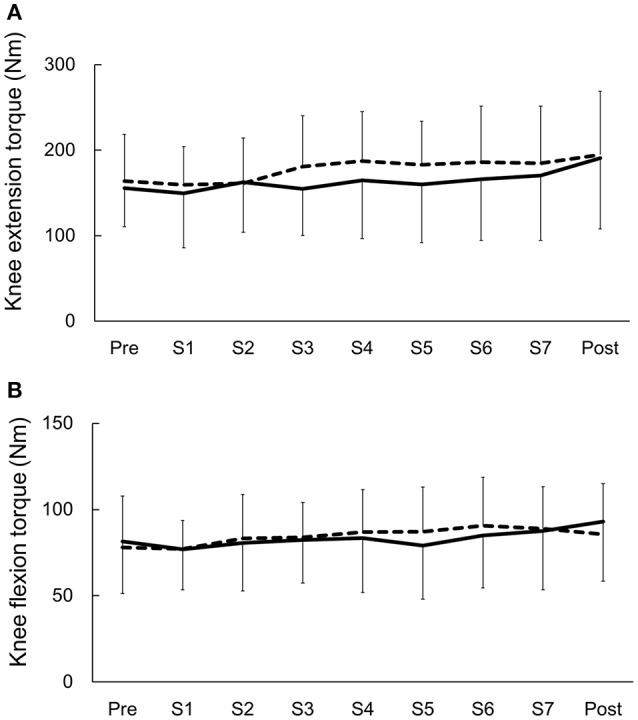
**Changes in knee torque before (Pre) and after (Post), and throughout the training sessions**. Data are presented as the mean ± standard deviation. Solid lines represent the anodal transcranial direct current stimulation (tDCS) group. Dashed line represents the sham tDCS group. **(A)** Effects of anodal tDCS (real and sham) combined with muscle strength training on knee extensor torque. **(B)** Effects of anodal tDCS (real and sham) combined with muscle strength training on knee flexor torque.

Peak torques of knee extensors and flexors in the anodal and sham tDCS groups are shown in Table 2. Changes in peak torques before and after, and throughout the training sessions are shown in Figures 3, 4. The percentages of those who showed clinically meaningful gain in muscle strength, as determined by the reported percentage of the smallest real difference (18 and 19% for knee extension and flexion, respectively; Sole et al., 2007) were as follows. Those who had a clinically meaningful gain in knee extensor torque included 10 of 12 participants (83.3%) and 7 of 12 participants (58.3%) in the anodal group and sham group, respectively. Those who had a clinically meaningful gain in knee flexion torque included 5 of 12 participants (41.7%) and 3 of 12 participants (25.0%) in the anodal group and sham group, respectively. Primary outcome results of the 2-way repeated ANOVA showed a statistically significant main effect of time [*F*_(1, 22)_ = 6.758, *p* = 0.016, $\eta_{p}^{2}$ = 0.235, MSe = 13005.375] on extensor muscle strength of the intervention side, whereas main effects for the intervention [*F*_(1, 22)_ = 0.063, *p* = 0.804, $\eta_{p}^{2}=$ 0.003, MSe = 429.005] and intervention × time interaction [*F*_(1, 22)_ = 0.032, *p* = 0.860, $\eta_{p}^{2}=$ 0.001, MSe = 61.427] were not observed. Results of *post-hoc* analysis showed that knee extensor strength was significantly increased after the intervention compared to before the intervention (*p* = 0.016). Regarding flexor muscle strength on the intervention side, a significant main effect was detected for time [*F*_(1, 22)_ = 10.485, *p* = 0.004, $\eta_{p}^{2}\;=$ 0.323, MSe = 1108.802] but not for the intervention [*F*_(1, 22)_ = 0.199, *p* = 0.660, $\eta_{p}^{2}\;=$ 0.009, MSe = 364.652] or intervention × time interaction [*F*_(1, 22)_ = 0.463, *p* = 0.503, $\eta_{p}^{2}\;=$ 0.021, MSe = 49.005]. Results of *post-hoc* analysis demonstrated that knee flexor strength was significantly increased after the intervention compared to before the intervention (*p* = 0.004).

**Table 2:** **Peak knee extensor and flexor torque**.

	**Anodal tDCS group (*****n*** = **12)**		**Sham tDCS group (*****n*** = **12)**	
	**Pre mean (*SD*)**	**Post mean (*SD*)**	**Pre mean (*SD*)**	**Post mean (*SD*)**
**KNEE EXTENSOR TORQUE (Nm)**				
Intervention side	155.9 (45.3)	191.1 (83.2)	164.1 (54.7)	194.8 (74.1)
Non-intervention side	162.9 (56.0)	170.3 (68.6)	159.6 (53.0)	165.9 (51.2)
**KNEE FLEXOR TORQUE (Nm)**				
Intervention side	81.5 (30.3)	93.1 (34.6)	78.0 (29.8)	85.6 (29.5)
Non-intervention side	81.8 (27.9)	88.8 (30.0)	86.3 (25.8)	84.3 (24.4)

*Data show the muscle strength peak torque before (Pre) and 3 weeks after training (Post)*.

Results of 2-way repeated ANOVA showed no significant main effect of time [*F*_(1, 22)_ = 0.413, *p* = 0.527, $\eta_{p}^{2}=$ 0.018, MSe = 564.441], intervention [*F*_(1, 22)_ = 0.033, *p* = 0.858, $\eta_{p}^{2}=$ 0.001, MSe = 173.280], or intervention × time interaction [*F*_(1, 22)_ = 0.003, *p* = 0.958, $\eta_{p}^{2}<$ 0.001, MSe = 3.853] on the knee extensor of the non-intervention side. Concerning the knee flexor on the non-intervention side, there was no significant main effect of time [*F*_(1, 22)_ = 0.736, *p* = 0.400, $\eta_{p}^{2}=$ 0.032, MSe = 73.508], intervention [*F*_(1, 22)_ = 0.000, *p* = 0.998, $\eta_{p}^{2}<$ 0.001, MSe = 0.007], or intervention × time interaction [*F*_(1, 22)_ = 2.387, *p* = 0.137, $\eta_{p}^{2}=$ 0.098, MSe = 238.521].

Results of secondary outcome measure showed a significant main effect for time [*F*_(1, 22)_ = 5.017, *p* = 0.003, $\eta_{p}^{2}=$ 0.186, MSe = 0.908] but not for the intervention [*F*_(1, 22)_ = 0.349, *p* = 0.561, $\eta_{p}^{2}=$ 0.016, MSe = 0.087] or intervention × time interaction [*F*_(1, 22)_ = 0.866, *p* = 0.466, $\eta_{p}^{2}=$ 0.038, MSe = 0.233] for extensor muscle strength on the intervention side (Figure 4A). Regarding flexion muscle strength on the intervention side, there was a significant main effect of time [*F*_(1, 22)_ = 3.681, *p* = 0.009, $\eta_{p}^{2}\;=$ 0.143, MSe = 0.854], whereas main effects for the intervention [*F*_(1, 22)_ = 0.015, *p* = 0.903, $\eta_{p}^{2}\;=$ 0.001, MSe = 0.052] and intervention × time interaction [*F*_(1, 22)_ = 1.171, *p* = 0.329, $\eta_{p}^{2}=$ 0.051, MSe = 0.346] were not observed (Figure 4B). Results of *post-hoc* analysis did not show any differences between the training sessions *(p* > 0.05).

## Discussion

In the present study, the repeated sessions of lower extremity strength training combined with tDCS did not produce significantly greater increases in strength than the sessions of training with sham tDCS. We believe that the null result is relevant to avoid unsuccessful clinical trials and minimize publication bias.

The present study used a strict experimental design, namely a triple-blind sham-controlled design, to exclude as many potential confounders as possible (e.g., experimenter expectations regarding the intervention outcome). Therefore, we believe that the present result includes minimum experimental bias. To the best of our knowledge, the present study is the first to investigate the effects of tDCS on muscle strength using a triple-blind procedure.

Hendy and Kidgell (2013) examined the effect of 3-week upper extremity strength training combined with tDCS. They found that the significant strength gains were only marginally greater in the anodal tDCS group than in the sham group after the intervention (14.9 and 11.2%, respectively). However, they found no significant difference in the increases in strength between the groups. The present result is in agreement with their finding. Together with the finding by Hendy and Kidgell (2013), the effects of repeated tDCS combined with muscle strength training may not modify muscle strength augmentation in healthy individuals.

However, we should note that the present study is the first feasibility study to examine the effect of repeated tDCS on the lower leg muscle strength. Clearly, further studies using alternative tDCS (e.g., different electrode configuration, size, or current intensity) and training protocols (eccentric vs. concentric training) are needed. The necessity of further studies with different parameters is obvious from the studies that have evaluated the acute effects of tDCS on lower extremity muscle strength. Washabaugh et al. (2016) reported that anodal tDCS combined with motor task produced greater knee extension torques relative to sham compared with anodal tDCS alone. In contract, among other tDCS studies, this first triple-blind study provides cogent evidence that the enhancement of knee torque was not seen with anodal tDCS combined with muscle strength training during each training session, compared to the sham tDCS with muscle strength training (Figure 4). However, the acute effects are inconsistent across studies (Tanaka et al., 2009, 2011; Montenegro et al., 2015, 2016; Angius et al., 2016; Washabaugh et al., 2016) and seem dependent on tDCS protocols, training tasks, muscle groups, and subject populations. In this context, computational modeling would be useful to understand the different spatial distributions of the electric field induced by different tDCS protocols (electrode configuration, size, or current intensity; Laakso et al., 2015, 2016).

Anodal tDCS was expected to alter the motor unit recruitment and to increase the descending drive even during muscle strength training (Krishnan et al., 2014). However, the additional strength gains by repeated tDCS were not obtained in this study. This finding indicates that the effects on motor recruitment strategies induced by anodal tDCS were relatively small compared with physiological adaptation in healthy individuals. However, patients with stroke had a decreased number of functioning motor units (McComas et al., 1973) and decreased firing frequency of the motor units (Rosenfalck and Andreassen, 1980; Tang and Rymer, 1981). Hummel et al. (2006) applied anodal tDCS to stroke patients and found that the reaction times and pinch forces improved in patients with relatively severe impairments who were unable to perform skilled ADL-like motor tasks compared to those in stroke patients with mild impairments. Therefore, it is likely that the use of tDCS is more effective in stroke patients who exhibit decreased motor cortex excitability. Future studies should investigate the effects of lower extremity muscle strength training combined with tDCS in stroke patients.

There are some limitations to this study. The first limitation is the small sample size. Twelve subjects per group seem underpowered, although 12 is close to the sample size (15 subjects per group) of Hendy and Kidgell's (2013) study. There is a large inter-individual variation in the outcome of tDCS over the hand motor cortex (Wiethoff et al., 2014; Laakso et al., 2015, 2016), with approximately one-half of subjects failing to respond to the stimulation in the expected manner (Wiethoff et al., 2014). Recently, such a large inter-individual variation was also observed in tDCS over the leg motor cortex (Madhavan et al., 2016; van Asseldonk and Boonstra, 2016). As the percentage of those who had a clinically meaningful effect was larger in the tDCS group than in the sham group, it is possible that an additive effect of muscle strengthening may exist in some individuals in the tDCS group. However, the small sample size might not be able to detect the additive effect of tDCS on muscle strength training by the large variability of the effects of tDCS among participants. Therefore, a large inter-individual variation in the outcome of tDCS may contribute to our negative results at a group level. Future studies with a large sample size are needed to clarify this point. The second limitation is that the neurophysiological data regarding the effects of muscle strength training combined with anodal tDCS are lacking in this study. Therefore, the neurophysiological activity after muscle strength training combined with tDCS should be examined in future using TMS and/or neuroimaging techniques.

## Conclusions

Results of the present study showed that repeated anodal tDCS does not enhance the effects of lower extremity muscle strength training in healthy individuals. However, it is insufficient to draw firm conclusions based on the present null findings. Future studies should focus on understanding the conditions that induce muscle strength with tDCS.

## Author contributions

TY and ST conceived and supervised the study. KM, TY, ST, and YO designed the experiments. KM, TT, and KK carried out the experiments. KM and ST analyzed the data. KM, TY, YO, and ST wrote the manuscript. All authors approved the final version of the submitted manuscript.

### Conflict of interest statement

The authors declare that the research was conducted in the absence of any commercial or financial relationships that could be construed as a potential conflict of interest.
